# Glutamine metabolism, a double agent combating or fuelling hepatocellular carcinoma

**DOI:** 10.1016/j.jhepr.2024.101077

**Published:** 2024-03-27

**Authors:** Razan Abou Ziki, Sabine Colnot

**Affiliations:** 1 INSERM, Sorbonne Université, Centre de Recherche des Cordeliers (CRC), Paris, F-75006, France; 2 Équipe labellisée Ligue Nationale Contre le Cancer, France

**Keywords:** Hepatocellular carcinoma, beta-catenin, glutamine metabolism, glutaminase, glutamine synthetase, liver zonation

## Abstract

The reprogramming of glutamine metabolism is a key event in cancer more generally and in hepatocellular carcinoma (HCC) in particular. Glutamine consumption supplies tumours with ATP and metabolites through anaplerosis of the tricarboxylic acid cycle, while glutamine production can be enhanced by the overexpression of glutamine synthetase. In HCC, increased glutamine production is driven by activating mutations in the *CTNNB1* gene encoding β-catenin. Increased glutamine synthesis or utilisation impacts tumour epigenetics, oxidative stress, autophagy, immunity and associated pathways, such as the mTOR (mammalian target of rapamycin) pathway. In this review, we will discuss studies which emphasise the pro-tumoral or tumour-suppressive effect of glutamine overproduction. It is clear that more comprehensive studies are needed as a foundation from which to develop suitable therapies targeting glutamine metabolic pathways, depending on the predicted pro- or anti-tumour role of dysregulated glutamine metabolism in distinct genetic contexts.

**Key points**

• Rewiring glutamine metabolism is a key event impacting HCC tumorigenesis.
• Glutamine-consuming HCCs belong to the “high proliferative” class of HCC.
• Glutamine-producing HCCs belong to the “low proliferative” β-catenin class of HCC.
• Glutaminase inhibitors are used for the treatment of glutamine-consuming HCCs.
• No consensus exists on the use of glutamine modulators for the treatment of glutamine-producing HCCs.
• New therapeutic strategies based on glutamine vulnerability of HCCs will require mutational characterisation and a multi-drug approach.
• Increasing knowledge of the pro- *vs.* anti-tumoral effects of glutamine anabolism and catabolism is a fundamental requirement.

## Introduction

Metabolic reprogramming is now a hallmark of cancer.^1^ Translational research has in turn seized on the therapeutic potential of targeting tumour metabolism. Otto Warburg founded this field almost a century ago.^2^ He described how malignant cells increase glucose uptake and glycolysis rather than oxidative phosphorylation, in a process independent of oxygen availability. Referred to as the “Warburg effect”, this discovery paved the way for tumour imaging through PET/CT using radiolabelled ^18^F-deoxyglucose, based on the addiction of tumours to glucose. Nevertheless, studies in recent years have observed the extraordinary metabolic plasticity of tumour cells, which greatly exceeds the Warburg effect. This plasticity is crucial in tumour initiation, progression, and resistance to anti-tumour treatment, working in a cell-autonomous manner or by interacting with the tumour microenvironment and microbiome. Among the metabolic pathways implicated in this phenomenon, the metabolism of amino acids changes in tumours facing adaptative requirements. This last decade has seen renewed research into the metabolism of glutamine (Gln) in cancer, and notably in HCC. Glucose and Gln are known as essential for the proliferation of tumour cells in culture.^3^ Recent discoveries have found that Gln is required to replenish tricarboxylic acid (TCA) cycle intermediates (so-called “anaplerosis”), which is crucial for the production of ATP.^4^ It also plays a key role in the metabolism of major macromolecules in mammalian cells. Consequently, tumour growth is most likely impacted by glutamine metabolism.

## Glutamine metabolism

Gln is the most abundant amino acid found in the body.^5^ Though classified as a non-essential amino acid, it is required for cell proliferation in culture. At certain periods in the human life cycle, it is essential, and may be required in higher quantities. Such periods include pregnancy,^6^ breastfeeding,^7^^,^^8^ and recovery from trauma or malignancy.^9^^,^^10^

**Fig. 1:**
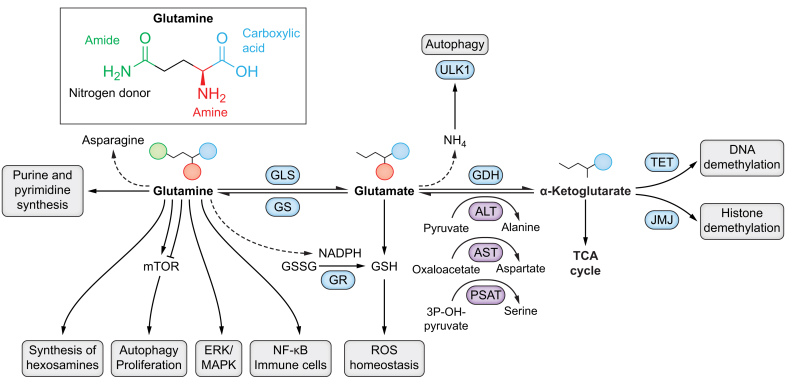
Multiple roles of glutamine and its derivatives in mammalian cells. See the text for further details. Upper panel: the structure of glutamine. Lower panel: multiple roles of glutamine, glutamate and αKG. The aminotransferase reactions are shown in purple. αKG, alpha-ketoglutarate; ALT, alanine aminotransferase; AST, aspartate aminotransferase; ERK, extracellular signal-regulated kinases; GS, glutamine synthetase; GLS, glutaminase; GDH, glutamate dehydrogenase; GR, glutathione reductase; GSSG, glutathione disulfide; GSH, glutathione; JMJ, Jumonji C (JmjC) domain-containing; MAPK, mitogen-activated protein kinase; mTOR, mammalian target of rapamycin; PSAT, phosphoserine amino-transferase; ROS, reactive oxygen species; TET, ten eleven translocation; ULK1, Unc-51-like autophagy-activating kinases 1.

Its unique structure sets it aside from other amino acids, as it contains an amino group in the α position and an amide group (in the γ position) (Fig. 1). It serves as a nitrogen and carbon donor, and transports ammonia in the body, thus playing an important role in nitrogen balance and pH homeostasis.^11^^,^^12^ It is involved in protein and glucose synthesis, as well as being incorporated into other amino acids. Gln mediates a variety of biochemical processes which involve its own amide group: this is the case for the synthesis of purines and pyrimidines, of asparagine,^13^ and of hexosamines.^14^ Glutaminolysis gives rise to glutamate (Glu, an essential amine donor), which is involved in glutathione production and anti-oxidant response,^15^ and to α-ketoglutarate (αKG, also known as 2-oxoglutarate), through which Gln plays the role of replenisher of the TCA cycle.^4^ When entering the TCA cycle to form malate, Gln is converted into pyruvate, leading to the reduction of NADP to NADPH (Fig. 1).

The rates of glutamine production and consumption are not homogeneous throughout the body, but are instead organ- and tissue-specific, with specific transporters required for its cell efflux and influx. For instance, the small intestine, leukocytes and kidneys are Gln-consuming tissues with high glutaminase (GLS) activity, where Gln serves as a substrate for ammonia genesis.^16^^,^^17^ On the other hand, Gln is highly synthesised in skeletal muscles.^5^ Gln metabolism can also vary within the same tissue according to different metabolic needs. This phenomenon is mostly seen in the lungs, liver, brain, skeletal muscles, and adipose tissues. While muscles normally store up to 80% of the body’s total Gln, they can reduce Gln synthesis under certain conditions, such as injury, catabolic stress, and shortage of nutrients.^18^ In the liver, Gln also plays an important role in osmoregulation and, consequently, in biliary excretion.^19^ In sepsis, the liver becomes the principal organ implicated in Gln uptake.^20^

In tissues, Gln synthesis is catalysed by the action of the glutamate ammonia ligase enzyme, also known as glutamine synthetase (GS). It uses ATP to form Gln from Glu and ammonia ions (NH4+).^21^ The reversed reaction is hydrolysed by GLS enzymes. Glu serves as a substrate for hepatic gluconeogenesis, urea synthesis, renal ammonia genesis, and acts as a significant fuel source for immune cells.^22^ Two types of glutaminase enzymes exist in mammals: GLS1, which is mainly expressed in the kidney (kidney-type), and GLS2, which is exclusively expressed in the liver (liver type).^23^ Glu is synthesised from αKG and NH4, by the action of the glutamate dehydrogenase (GDH or GLUD), which in turn can catalyse the conversion of Glu to αKG and ammonia while reducing NAD(P)+ to NAD(P)H.^24^ αKG is an integral component of the TCA cycle. It can also be produced from Glu without producing ammonia through aminotransferase reactions catalysed by a group of aminotransferases/transaminases, such as aspartate aminotransferase (glutamate oxaloacetate transaminase), alanine aminotransferase (glutamate-pyruvate transaminase), and phosphoserine aminotransferases (Fig. 1).

Rarely mentioned in research, the alternative GTωA pathway also produces αKG from Gln after its transamination to α-ketoglutaramate, followed by ωA (ω-amidase)-catalysed hydrolysis of α-ketoglutaramate to αKG. This pathway is reviewed elsewhere,^25^ and must be taken into account whenever Gln addiction is being considered, in particular in the case of cancers.

## Glutamine consumption in cancer

Cancer cells are significant consumers of Gln. They compete with normal cells for available Gln and depend on it for growth and tumorigenesis.^21^ Gln deprivation has been shown to induce apoptosis in transformed cells.[26], [27], [28] Tumours display higher rates of glutaminolysis than normal tissues, exemplified by the high expression and activity of glutaminolytic enzymes such as GLS (especially GLS1), which is a therapeutic target in cancer.^29^ Increased Gln transport across the plasma membrane is also a signature of Gln addiction. It involves high expression of Gln transporters, such as SLC38A5 and SLC6A14, for its uptake, or SLC1A5 for its efflux or influx.^30^ By contrast, only a small number of studies have focused on enhanced tumour Gln synthesis through GS overexpression. This aspect will be treated in the following paragraphs. Gln use is well documented in different types of cancers, including cancers of the liver (treated below), breast,^31^^,^^32^ ovaries,^33^ pancreas,[34], [35], [36] blood (leukaemia),^37^^,^^38^ brain,^39^ and kidney.^40^ It has been shown that Gln supplementation or GLS inhibition attenuates tumorigenesis and improves the state of patients undergoing chemotherapy.

Research has demonstrated Gln’s role as a multifunctional amino acid which participates in several metabolic, signalling, and epigenetic pathways during tumour initiation, progression and treatment. In this paper, we will give details of the pathways that have been the subject of most studies so far.

### Glutathione

Glutathione (GSH) is an anti-oxidant tripeptide, synthesised from the amino acids Glu, cysteine, and glycine.^41^ In general, Gln-derived Glu first combines with cysteine in an ATP-dependent manner to form γ-glutamylcysteine, which then combines with glycine to form GSH. Gln also regulates oxidative homeostasis and glutathione synthesis by supplying NADPH, which reduces glutathione disulfide, an oxidized form of GSH, to GSH via the enzyme glutathione reductase^42^^,^^43^ (Fig. 1). Given that glutathione neutralises peroxide free radicals and eliminates excessive reactive oxygen species (ROS), its levels are critical in cancer cells. For example, GSH has been shown to be essential for tumour initiation in breast cancer.^44^ Glutathione levels are elevated in HCC and correlate with cell growth rate.[44], [45], [46] GSH also promotes HCC formation and desensitises cells to the tumour-suppressive effects of sorafenib.^47^ Alternatively, the disruption of liver mitochondrial pyruvate carrier prevents HCC development by reprogramming Gln to enter the TCA cycle rather than produce glutathione, thus inducing apoptosis.^48^

### Autophagy and mTOR pathway

Many processes involved in Gln metabolism directly or indirectly regulate autophagy. However, it remains unclear whether Gln is an activator or a repressor of autophagy. For instance, Gln can inhibit ROS-induced autophagy through GSH synthesis. On the other hand, adding increasing concentrations of Gln has been shown to upregulate basal autophagy in intestinal epithelial cells.^49^ The underlying mechanisms have been hypothetically linked to Gln’s ability to regulate mammalian target of rapamycin (mTOR), which inhibits autophagy^50^ (Fig. 1). Gln has been shown to be crucial for essential amino acid- and growth factor-induced mTOR activation in Hela cells.^51^ In this study, the addition of growth factor-rich serum led to increased intracellular concentrations of Gln, via uptake through SLC1A5. This intracellular Gln was then used as an efflux substrate for the uptake of extracellular leucine by the bidirectional transporter SLC7A5/SLC3A2, leading to the activation of mTOR complex 1 (mTORC1) and inhibition of autophagy.

### Other signalling pathways

Gln also modulates the ERK/MAPK pathway (Fig. 1), which is deregulated in HCC, and which is a target of sorafenib. ERK has been shown to regulate Gln uptake to activate T cells.^52^ It interacts with MEK and ELK1 (ETS like-1 protein) to activate GDH1 (glutamate dehydrogenase 1) in response to epidermal growth factor receptor signalling in glioblastoma.^53^ Gln is also essential for epidermal growth factor-mediated intestinal epithelial cell proliferation via the ERK/c-Jun-N-terminal kinase pathway.^54^^,^^55^ In studies performed both *in vitro* and on human HCC, severe metabolic alterations of HCC cells have been correlated with ERK pathway activation. Gln deprivation causes deregulated metabolism, ERK pathway activation, and increased proliferation, as well as mediating resistance to kinase inhibitors. Blocking the ERK pathway in cells with severe metabolic stress renders them more sensitive to treatment with kinase inhibitors.^56^ Gln has also been implicated in pathways such as apoptosis^57^ and immune modulation through the NF-kB pathway. The NF-κB subunit p65 regulated GLS1 expression in HCC cells and impacted their proliferation. p65-GLS1 deregulation has also been associated with poor prognosis in patients with HCC.^58^

### Epigenetic regulation through αKG

Apart from its metabolic role, Gln may participate in epigenetic regulation of DNA and histones through glutaminolysis-mediated αKG. When entering the TCA cycle, αKG is converted to malate, pyruvate and, later, to lactate through the action of lactate dehydrogenase.^59^^,^^60^ The epigenetic power of αKG resides in its ability to act as a cofactor for several chromatin-modifying enzymes, like the ten eleven translocation (Tet) and Jumonji C domain-containing (JMJD) families of demethylases^59^^,^^61^ (Fig. 1). The tumour suppressor TET2 oxidises 5-methylcytosine to 5-hydroxymethylcytosine, and subsequently to 5-formylcytosine and 5-carboxylcytosine, ultimately facilitating DNA demethylation.^62^ In many cancers, loss of TET expression results in an overall genomic reduction of 5-hydroxymethylcytosine, which can explain the hypermethylation of gene promoters seen in HCC.^63^ JMJD demethylases involve histone lysine demethylase activity,^64^ and require the involvement of oxygen and αKG as cofactors, which makes them sensitive to metabolic changes within cells.^65^ Investigations into the role of JMJD4 alterations in inflammatory diseases and cancers are ongoing. A recent study showed that JMJD4 demethylates retinoic acid-inducible gene-I to prevent necroinflammation and NASH (non-alcoholic steatohepatitis)-induced hepatocarcinogenesis.^66^ In this context, it is relevant to note that abnormal αKG anabolism can generate oncometabolites: isocitrate dehydrogenase 1 and 2 (IDH1/2) are enzymes that normally catalyse the interconversion of isocitrate and αKG. IDH1/2 are frequently mutated in cancers, but not in HCC, and the gain-of-function IDH1/2-mutantenzymes lead to the accumulation of the oncometabolite D-2-hydroxyglutarate, resulting in aberrant DNA and histone methylation by competitively inhibiting enzymes such as TET and JMJD.^67^^,^^68^

### Immunity

Gln is known to be an immune fuel,^69^ given that a shortage of Gln inhibits proliferation of and cytokine production by T lymphocytes.^52^ Moreover, GS has been shown to be constitutively expressed in liver macrophages and to regulate the process of inflammation by modulating macrophage differentiation.^70^^,^^71^ GS inhibition has shown the capacity to promote macrophage switching back towards a M1-like phenotype, and thereby to markedly reduce metastasis. Gln metabolism is important for tumour signalling with its microenvironment, thereby impacting the efficiency of immunotherapy. Tumour cell-specific knockdown of GLS improved anti-tumour T cell activation and response to immunotherapy in breast cancer.^72^ In another study, high-mobility group box 1 (HMGB1) promoted Gln metabolism, thus inducing stemness and tumorigenesis in HCC cells.^73^ HMGB1 has previously been shown to be a crucial cytokine in certain diseases, including non-alcoholic fatty liver disease and HBV-related HCC. It has also been described as a “nuclear weapon” in immunity. Suppressing the expression of HMGB1 could reduce Gln metabolic activity and enhance the response of HCC cells to programmed death-ligand 1 antagonists. Moreover, TCGA (The Cancer Genome Atlas) and single-cell RNA-sequencing data suggests that the tumour-killing ability of the CD8 T subpopulation can be inhibited when Gln metabolism is higher in tumour cells compared to CD8 T cells.^74^ Synchronised abrogation of Gln metabolism in tumour cells and CD8 T cells enhances the tumour-infiltrating capacity of CD8 T cells and the efficacy of immunotherapy in HCC cells.

## Pathogenesis of HCC

World Health Organization projections estimate that one million people will die annually from primary liver malignancies by 2030.^75^ HCC accounts for more than 90% of liver cancers. Its poor prognosis is generally due to late diagnosis and unsatisfactory treatment. Surgical resection and liver transplantation are performed in less than one-third of patients, and even these interventions are frequently followed by tumour recurrence. Patients with advanced cancers have long been treated with tyrosine kinase or multi-kinase inhibitors, among which sorafenib, a multitargeted tyrosine kinase inhibitor, has proven efficient in cases of HCC. Kinase inhibitors or anti-angiogenic treatments are now combined with immune checkpoint inhibitors to provide patients with advanced HCC with an alternative first-line or second-line therapy, improving their life expectancy.^76^

**Fig. 2:**
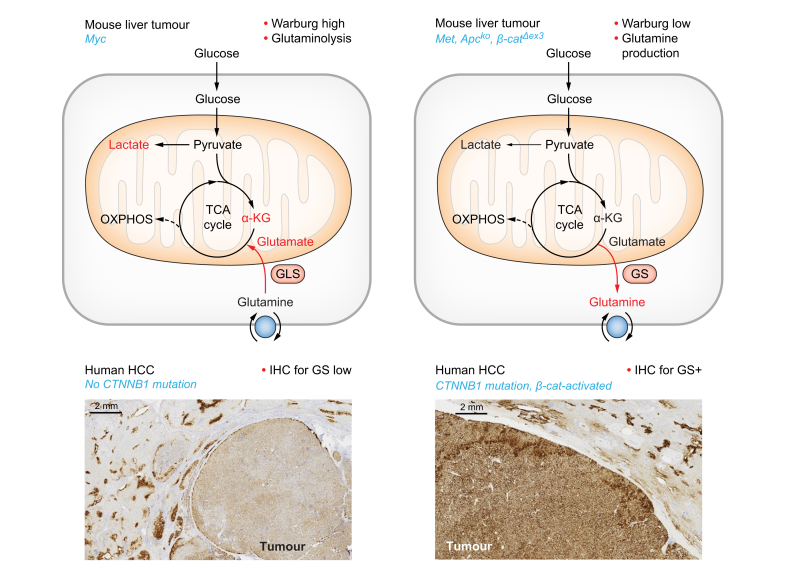
Two distinct types of glutamine metabolism occur depending on the genetic context of HCC in mice and humans. Upper panel: mouse liver tumours are characterised either by a strong Warburg effect and glutaminolysis when they depend on Myc overactivation,^79^ or by undetectable Warburg effect and strong glutamine synthesis when they depend on Met, Apc loss or *CTNNB1* deletion of exon 3.^85^^,^^87^ Lower panel: human HCCs with β-catenin mutational activation are characterised by their strong immunostaining (IHC) for GS, which constitutes a biomarker of these cancers. αKG, alpha-ketoglutarate; GLS, glutaminase; GS, glutamine synthetase; HCC, hepatocellular carcinoma; IHC, immunohistochemistry; OXPHOS, oxidative phosphorylation; TCA, tricarboxylic acid.

HCCs mostly develop on a cirrhotic background, and are associated with chronic liver disease deriving from a known aetiology: chronic viral hepatitis (C or B), alcohol abuse, or metabolic dysfunction-associated steatohepatitis (MASH, formerly known as NASH).^76^^,^^77^ The main pathways deregulated through mutations are related to telomere maintenance (mainly mutations in the *TERT* promoter), β-catenin signalling activation (mainly *CTNNB1* mutations), cell cycle regulation (mainly *TP53* mutations), epigenetic modifications (mainly *ARID1A* mutations), and others.^78^ Molecular classifications, which include aetiological, histological, immunological, genetic, epigenetic and metabolic features, have helped to further elaborate and refine new therapies. So far, two main classes have emerged. The first is characterised by enriched *TP53* mutations, with strongly proliferative tumours, a subset of which possess stem cell features. The second class is characterised by *CTNNB1* mutations, by immune exclusion and by a specific metabolism involving cholestasis and the overexpression of GS (Fig. 2).

## Metabolic rewiring of HCC: Towards glutaminolysis or glutamine synthesis

In a pioneering study, Yuneva *et al.* demonstrated definitively that the metabolic profile of tumours depends on both tissue type and on the genetic alterations responsible. They analysed lung and liver tumours generated after activation of Myc or Met (Fig. 2).^79^ They found that Myc-activated tumours in the liver were merely glycolytic, with an associated increase in glutaminolysis, whereas Met-activated tumours over-synthesised Gln, due to an overexpression of GS. Below, we describe these two types of tumour metabolism as corresponding to the two classes cited above: a highly proliferative one, which would be merely glutaminolytic, and a less proliferative one, characterised by its production of Gln and its *CTNNB1* mutation status.

### Glutaminolytic GLS1^high^ HCC

Previous studies strongly associated cancers involving *TP53* mutation, *MYC* amplification and c-JUN oncogene overexpression with an overexpression of GLS1, which favours glutaminolysis.[80], [81], [82], [83] Such cancers behave like mouse Myc liver tumours, being both glycolytic and glutaminolytic. Compared to its expression in normal tissues, GLS1 overexpression in HCC tissues has been correlated with late stage clinicopathological features. GLS1 has also been described as critical for HCC cell proliferation *in vitro* via activation of the AKT/GSK3β/CyclinD1 signalling pathway.^23^ The effect of targeting GLS1 was to attenuate stemness by increasing ROS and inhibiting the Wnt/β-catenin pathway.^84^ In contrast to this, GLS2 seems to play an inhibitory role in cell growth in HCCs by negatively regulating the PI3K/AKT pathway (83).

### Gln synthesising GS^high^ HCCs are *CTNNB1*-mutated and β-catenin-activated

We previously found that the overexpression of GS in mouse HCC is a biomarker of β-catenin activation in these tumours.[85], [86], [87] GS is also a specific biomarker of strong β-catenin activation in human HCC with *CTNNB1* mutations (Fig. 2).[88], [89], [90], [91] We demonstrated that mouse β-catenin-activated livers are not addicted to glucose, from the observation that ^18^F-deoxyglucose-PET imaging is not enhanced in β-catenin-activated livers and tumours. We demonstrated that this is also the case in *CTNNB1*-mutated human HCCs.^92^^,^^93^ Together with Yuneva’s study,^79^ these observations clearly show that β-catenin-activated HCCs behave like mouse Met liver tumours, being Gln producers with no clear addiction to glucose.

## β-Catenin signalling reprogrammes liver and Gln metabolism

The particular features of β-catenin-activated HCC are reminiscent of the physiological role of β-catenin signalling in the liver, which is to orchestrate liver zonation.^94^^,^^95^ This dynamic geographical organisation of the liver allows hepatocytes to fulfil their metabolic functions, depending on their localisation along the porto-central axis. We previously showed that the Wnt/β-catenin pathway is activated in the pericentral area, and is responsible for bile synthesis, glycolysis, Gln synthesis and xenobiotic metabolism.^86^^,^^96^ Two zonated processes of ammonia clearance occur in the liver. Either the ammonia enters the urea cycle for excretion, which occurs in the periportal zone surrounding the portal vein, or ammonia is assimilated into Glu and subsequently Gln.[97], [98], [99], [100], [101] The urea cycle enzymes (UCEs) and GLS2 are periportally expressed, whereas Gln synthesis takes place in the pericentral hepatocytes surrounding the central vein, collecting residual nitrogen waste or ammonia escaping urea synthesis.^97^ Gln synthesis in this zone acts as a “back-up system” for ammonia detoxification, ensuring that the excreted hepatic venous blood is free of toxic ammonia, which would otherwise cause lethal hepatic encephalopathy.[102], [103], [104]

*GLUL*, the gene coding for GS, is a major Wnt/β-catenin pericentral target gene.^96^^,^^105^^,^^106^ β-Catenin acts as a cofactor of transcription when stabilised by a Wnt cascade or by *CTNNB1* mutations, and translocates into the nucleus to associate with its TCF4 partner. The TCF4/β-catenin complex then binds to Wnt response elements on the promoters of target genes, including *GLUL*,^86^ increasing their transcription.

**Fig. 3:**
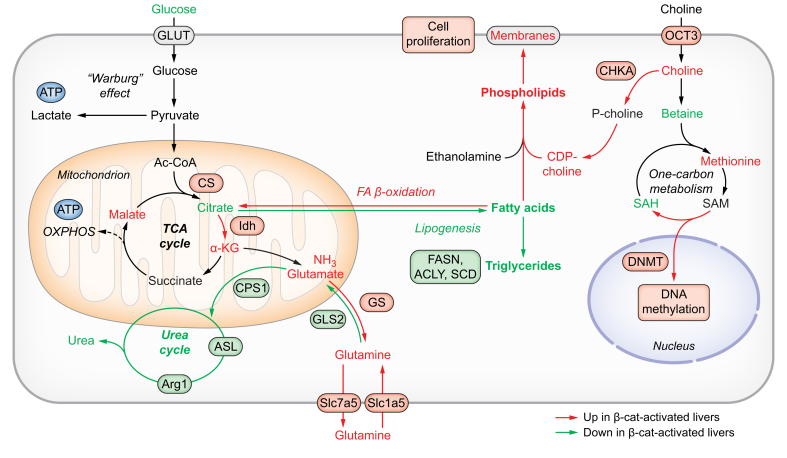
Metabolic reprogramming by β-catenin in the liver. Metabolomic and transcriptomic studies confirmed by functional assessments show that TCA cycle, glutamine transport and synthesis,^86^^,^^96^ fatty acid oxidation,^107^ choline uptake and use for phospholipid synthesis and DNA methylation,^92^ are enhanced by β-catenin signalling activation. Conversely, the urea cycle, glutaminolysis and lipogenesis are decreased.^86^^,^^92^^,^^96^^,^^107^ Ac-CoA, acetyl-CoA; αKG, α-ketoglutarate; Cit, citrate; DNMT, DNA methyltransferase; GLS, glutaminase; Glu, glutamate; GLUT, glucose transporters; GS, glutamine synthethase; Idh, isocitrate dehydrogenase; Mal, malate; Met, methionine; OCT, organic cation transporters; SAH, S-adenosyl homocysteine; SAM, S-adenosyl methionine; Slc7a5, solute carrier family 7 member 5; Slc1a5, solute carrier family 1 member; Succ, succinate.

Our previous studies in mice showed that activating β-catenin in the whole lobule through the loss of adenomatous polyposis coli generates a pericentral-like liver, which overproduces Gln but loses its periportal function of detoxifying ammonia, resulting in severe hyperammonaemia and death.^96^ These β-catenin-activated livers profoundly reprogramme their metabolism with excessive αKG and bile acid synthesis, decreased lipogenesis, and increased choline uptake and use (Fig. 3). Lastly, β-catenin-activated HCCs are addicted to fatty acids, and the inhibition of fatty acid oxidation is a suitable therapeutic approach for *CTNNB1-*mutated HCCs (Fig. 3).^107^ Excessive Gln synthesis is thus not an isolated metabolic change and occurs in β-catenin-activated HCCs of both mouse and human origin.^86^^,^[90], [91], [92]^,^^96^^,^^107^

## Is glutamine metabolism pro-oncogenic or tumour suppressive in β-catenin-mutated HCC?

**Fig. 4:**
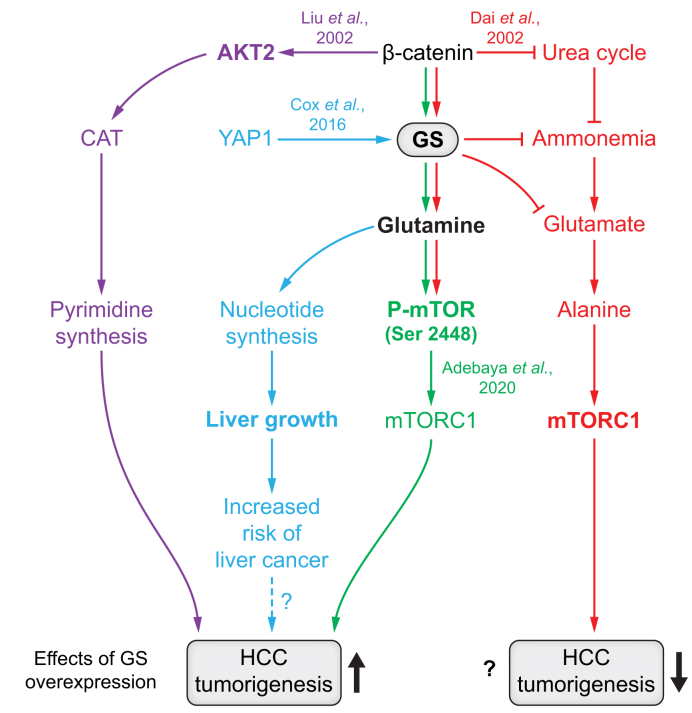
GS overexpression occurs in a context of β-catenin-mutated HCC and has pro-tumoral or anti-tumoral effects. Graphic summary of four contradictory publications discussed in the text. In purple, the data of Liu *et al.*, 2022; in blue, Cox *et al.*, 2016; in green, Adebayo *et al.*, 2019; in red, Dai *et al.*, 2022. GS, glutamine synthetase; HCC, hepatocellular carcinoma; mTORC1, mammalian target of rapamycin complex 1.

While the use of GS as a marker of Wnt/β-catenin-activated HCCs is widely known, the exact impact of Gln synthesis *vs.* Gln consumption in such tumours has yet to be clearly identified. An overview of the current hypotheses regarding this process are shown in Fig. 4.

In 2019, Adebayo *et al.* suggested that GS may have a pro-tumoral role in β-catenin-mutated HCCs via a cross talk with the mTORC1 pathway, and that the inhibition of GS-derived mTORC1 decreases tumorigenicity.^108^ mTOR offered considerable potential as a Gln partner, given that it can be regulated by the bidirectional transport of leucine and Gln across the membrane.^51^ The authors used an *in vivo* approach in mice. By hydrodynamic tail vein injection and in a stable manner, they delivered activating mutant β-catenin (S45Y, S33Y, Δ90), together with either c-Met (Met-β-catenin model) or Ras activation (Ras-β-catenin model) in the liver. β-Catenin-activated HCCs developed and expressed high levels of GS and Gln. The study described a positive correlation between GS expression and p-mTOR-s2448 in multiple genetic mouse models and human HCCs. As this suggested that p-mTOR-s2448 is an indicator of mTORC1 activation, they concluded that the presence of GS activates mTORC1.

Dai *et al.* recently provided evidence of GS’s tumour suppressor role in β-catenin-activated HCCs.^109^ The authors used an *in vivo* approach in mice similar to that described above. They delivered c-Met and the constitutively active mutant of β-catenin (ΔN90-β-catenin) into livers, either wild-type or harbouring hepatocyte-specific ablation of GS (GS^KO^). While their results confirmed the positive correlation between p-mTOR-s2448 and GS, they also showed that p-mTOR-s2448 is not correlated with mTORC1 activity in mice and human HCCs. In fact, while c-Met/β-catenin activation alone led to increased expression of phosphorylated 4EBP1 (T37/46, S65) and p-S6 (S235/235, S240/244), which are commonly used downstream effectors of mTORC1, a relatively greater increase was seen in the GS^KO^ livers compared to the WT livers. GS expression was inversely correlated with mTORC1 gene targets in WT livers and *CTNNB1*-mutated HCCs. *CTNNB1*-mutated patients with low GS expression showed poorer overall survival. These results suggest that GS actually suppresses mTORC1 activity, whereas mTORC1 activation induced by c-Met/β-catenin is independent of GS and leads to larger and more aggressive tumours. Although β-catenin-induced GS has been shown to render HCC cells sensitive to sorafenib by activating autophagy,^110^ Dai *et al.* did not find any difference in the expression of autophagy markers between GS^KO^ and WT mice after oncogenic activation. They also found no change in the expression of AKT, ERK, MAPK or AMPK. β-catenin activation was found to decrease the expression of UCEs in the liver.^86^^,^^96^ Dai *et al.* confirmed that c-Met/β-catenin activation causes a deregulated metabolism in mice marked by an increase in glycolytic and TCA cycle enzymes and decreased UCEs. Moreover, in *CTNNB1*-mutated patients, they found an inverse correlation between GS expression and the UCEs ARG1 (arginase 1), ASL (argininosuccinate lyase), and ASS1 (argininosuccinate synthetase). Given that GS upregulation normally helps the liver to compensate for a defective urea cycle, ammonia clearance was further impaired by the loss of GS, leading to hepatic encephalopathy, which has previously been shown to cause lethality in liver β-catenin-activated mice.^96^ In β-catenin-activated GS^KO^ mice, Gln levels decrease, whereas those of Glu and Glu-derived non-essential amino acids, especially alanine (Ala), increase, activating the mTORC1 pathway. The inhibition of alanine aminotransferase, which catalyses the transamination from Glu and pyruvate to Ala and αKG, with small-interfering RNA or two different inhibitors, led to decreased mTORC1 activation, and reduced the tumour burden of β-catenin-activated GS^KO^ mice. These findings revealed the underlying mechanisms behind the more deleterious phenotype observed in GS^KO^ mice and, possibly, in patients who express low GS in *CTNNB1*-mutated HCCs.

The complexity of the β-catenin-dependent HCC metabolome is described in a third study. Liu *et al.* used Cre-lox-mediated activation of β-catenin in the livers of HBV transgenic mice to induce tumour formation. In this setting, they showed that β-catenin activation increases pyrimidine synthesis independent of Gln synthesis (Fig. 4).^111^ This was surprising, as the first step of pyrimidine synthesis is mediated by the rate-limiting carbamoyl-phosphate synthetase 2 enzyme, whose substrate is Gln. Contrary to what might have been expected, β-catenin activated AKT2 kinase expression by directly binding to its promoter; AKT2 then phosphorylates and activates carbamoyl-phosphate synthetase 2. Relevant to these findings is the consideration of a known partner of β-catenin for purine/pyrimidine synthesis in HCC, namely YAP (yes-associated protein 1), a major regulator of organ size and tumorigenesis. Cox *et al.* show that YAP enhances GS expression and activity, thereby regulating nucleotide synthesis.^112^ Inhibiting GS either genetically or pharmacologically was sufficient to diminish nucleotide synthesis and tumour growth.

From this plethora of contradictory data, it appears that Gln overproduction can have either pro- or anti-tumoral effects. Thus, efforts to integrate Gln anabolism and catabolism into our understanding of the wider metabolic landscape seem more important than ever.

## Therapeutic opportunities

From the perspective of treating patients with cancer on a metabolic basis, several approaches have been investigated. One recent approach involves offering patients a specific diet which targets the main metabolic pathway involved in tumour growth.^113^ Amongst that strategy’s limitations are its reliance on the metabolic heterogeneity of the tumour and on the specific microbiome of patients with cancer.^114^ Another variable that must be considered is the patient’s overall condition, notably cancer cachexia, which is a frequent cause of death. This fact must be integrated into nutritional interventions designed to starve the tumour while strengthening the muscles of the patient.^115^ Besides dietary approaches, pharmacology remains at the forefront of current solutions for this issue.

Given the multiple direct and indirect functions exerted by Gln, it is likely that targeting its metabolism will efficiently improve HCC treatment. Several such therapeutic approaches already exist, such as the disruption of Gln transporters, GLS, GDH, and aminotransferase inhibitors.^29^ Many of those strategies have already shown favourable effects in HCC, and have been reviewed elsewhere.^116^

One obstacle that needs to be tackled when targeting Gln is the likelihood of increasing other Gln-related metabolites (like Glu, αKG and α-ketoglutaramate seen before), which can activate other tumorigenic pathways, thus leading to resistance. For this reason, a multi-drug approach has been shown to be more effective. For example, in mTOR inhibitor-resistant glioblastoma, simultaneous inhibition of mTOR and GLS leads to a synergistic increase in tumour cell death and growth inhibition in mice.^117^ In Gln-addicted HCC cells, a combinational treatment with CB-839 (glutaminase inhibitor) and V-9302 (an inhibitor targeting the ASCT2 transporter) produced better tumour inhibition than monotherapy with CB-839 alone. Combined therapy caused glutathione depletion and induced ROS, resulting in HCC cell apoptosis.^118^ Another therapeutic obstacle which must be addressed is tumour plasticity and its ability to compensate for the absence of a certain metabolite by shifting from one metabolic pathway to another. While Myc-induced mouse tumours seem to depend on the GLS1 isoenzyme to drive glutaminolysis, they surprisingly maintain Gln catabolism and incorporation into the TCA cycle after liver-specific knockout of *Gls1*.^119^ These tumours have been shown to overexpress GLS2 to compensate for GLS1 deletion. Alternatively, the possible involvement of the GTωA pathway in the compensation for liver Gls1^ko^ warrants further investigation.^25^ Myc-induced mouse tumours nonetheless managed to supply half of the Gln-derived carbons for the TCA cycle via amidotransferases, which utilise Gln as an amide nitrogen donor to produce Gln-derived Glu. Combined inhibition of glutaminases and amidotransferases was therefore necessary to reduce TCA cycle anaplerosis and amino acid biosynthesis, and thus suppress tumour cell proliferation. In that study, the inhibition of glutaminolysis was compensated for by increased glycolysis and the inhibition of fatty acid or serine biosynthesis was compensated for by increased dietary uptake.^119^ These results shed light on the need to apply a well-designed, multi-target approach that considers the complexity and versatility of Myc-induced liver tumours.

Due to the particular pattern of Gln-producing β-catenin-mutated HCCs, it is unlikely that these strategies would be efficient in that context. This underlies the need to stratify patients with HCC before treatment. For this, low invasive imaging might be an appropriate solution. We previously proposed ^18^F-fluoro-choline PET/CT imaging to delineate HCC with *CTNNB1* mutations.^92^ Alternatively, a specific MRI could be used, given that HCCs with *CTNNB1* mutations have shown a specific higher intensity in the hepatobiliary phase of Gd-EOB-DTPA (gadolinium ethoxybenzyl diethylenetriaminepentaacetic)-enhanced MRI, compared to the surrounding liver parenchyma.^120^ This is due to the enhancement by β-catenin of OATP1B3 expression, a Gd-EOB-DTPA transporter.^121^

To summarise, new therapeutic strategies based on the Gln vulnerability of HCCs will hinge on their mutational characterisation and likely on a multi-drug approach, which will, in turn, depend on an increased knowledge of the pro- *vs.* anti-tumoral effects of Gln anabolism and catabolism.

## Financial support

The team is funded by INSERM and the Ligue Nationale Contre le Cancer. RAZ’s salary is funded by a grant from the Institut National du Cancer INCa_1602.

## Authors’ contributions

RAZ and SC drafted the first version of the manuscript. Their writing was finalized by SC. The final revision was done by RAZ and SC.

## Conflict of interest

The authors of this study declare that they do not have any conflict of interest.

Please refer to the accompanying ICMJE disclosure forms for further details.
